# Pyramidal Lobe of the Thyroid Gland: Surgical Anatomy in Patients Undergoing Total Thyroidectomy

**DOI:** 10.1155/2015/384148

**Published:** 2015-07-07

**Authors:** Emin Gurleyik, Gunay Gurleyik, Sami Dogan, Utku Cobek, Fuat Cetin, Ufuk Onsal

**Affiliations:** ^1^Department of Surgery, Duzce University Medical Faculty, 81650 Duzce, Turkey; ^2^Department of Surgery, Haydarpasa Numune Education Hospital, 34668 Istanbul, Turkey

## Abstract

*Background*. Anatomic variations, the presence of the pyramidal lobe (PL), may impact completeness of thyroidectomy and effect of surgical treatment.* Method*. This study included 166 patients who underwent total thyroidectomy. The anterior cervical region between the thyroid isthmus and the hyoid bone was dissected during thyroid surgery. The incidence, size, and anatomical features of the PL were established in these patients.* Results*. The incidence of PL was 65.7%. No gender difference was found for PL incidence. The base of the PL was located at the isthmus in 52.3%, the left lobe in 29.4%, and the right lobe in 18.3% of patients. The mean length of the PL was 22.7 (range, 5–59) mm. The PL was longer than 30 mm in 23% of patients. One-third of the patients with short PL were men whereas women accounted for 80% of patients with long PL.* Conclusions*. The high incidence indicates that the PL is a common part of the thyroid. The PL generally originates from the isthmus near midline and is of variable length, extending from the isthmus up to the hyoid bone. Considering that the PL is a common structure, the prelaryngeal region should be dissected to achieve the completeness of thyroidectomy.

## 1. Introduction

At present, total thyroidectomy or hemithyroidectomy is procedures of choice for disorders of the thyroid gland requiring surgery. The completeness of resection has great importance, particularly for malignant diseases and autoimmune pathologies. Remnant tissue after surgical resection may complicate proper treatment of such diseases and the postoperative follow-up. Sometimes, anatomical variations of the gland can cause incomplete removal of the thyroidal tissue, which results in recurrent goitre formation. The presence of the pyramidal lobe (PL) is a typical example of anatomical variations affecting the completeness of thyroidectomy. The thyroid surgeon must have full knowledge of the anatomy of the thyroid gland, including all of its embryological, congenital, or acquired variations. The PL is defined as a thyroid tissue remnant of embryological origin located in the pretracheal region between the isthmus and the hyoid bone during intrauterine descent of the foetal lingual thyroid to its normal anatomical location. If present, neglecting excision of the PL generally prevents the completeness of thyroidectomy and may cause recurrent goitre.

In this prospective study, we aimed to determine the incidence and anatomical features of the PL in our patients who underwent thyroid surgery.

## 2. Material and Methods

This prospective study was conducted among 166 patients (mean age 48.5 (range 16–81) years, 125 (75.3%) females) who underwent total thyroidectomy for the surgical treatment of disorders of the thyroid gland between January 2013 and December 2014.

*Surgical Technique*. Full dissection of the lateral lobes was performed in a standard manner to achieve total resection of the gland. Both the lateral lobes were medially mobilized after complete surgical dissection. Before removal of the gland, the anterior cervical (pretracheal) region between the isthmus of the gland and the hyoid bone was completely visualised, observed, and explored for the presence of any thyroidal tissue, which, if present, was totally dissected from the isthmus up to the hyoid bone. Both of the lateral lobes, the isthmus, and the PL (if present) were totally excised to ensure the completeness of thyroidectomy.

The incidence of the PL was determined in patients who underwent total thyroidectomy.

The sex distribution of thyroidectomy cases with or without PL was established.

Another important point to be noted was the location of PL base originating from the main thyroid gland. We determined the originating point of the PL on thyroidectomy specimens.

The size and length of the PL were measured on fresh tissue immediately after the excision of the gland, and the PL was characterised as short (≤15 mm), medium (16–30 mm), or long (≥31 mm).

## 3. Results

Pyramidal lobes were found in 109 (65.7%) of 166 patients who underwent total thyroidectomy. No gender variation was observed for the incidence of PLs (male, 65.9%; female, 65.6%; Table 1). 82 of the 109 (75.2%) patients who had PLs were female.

In 52.3% of patients, the PL originated from the isthmus, followed by origin from the left side. Compared with 7.4% of male patients with PLs, the base of the PL was located on the right side in 22% of the 82 female patients with PLs. The PL originated from the isthmus in 48.8% and 63% of female and male patients, respectively (Table 2).

The mean length of the PL was 22.7 (range 5–59) mm in our present series, and 23% of the PLs were longer than 30 mm. Women constituted 80.6% of patients with PLs longer than 16 mm. The proportion of men was relatively higher (33.3%) among patients who had short PLs (Table 3).

**Figure 1:**
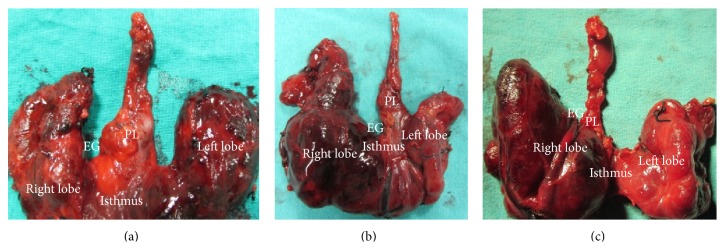
Long pyramidal lobe (PL): (a) originating from the isthmus at the midline, (b) originating from the left lobe, and (c) originating from the right lobe.

**Figure 2:**
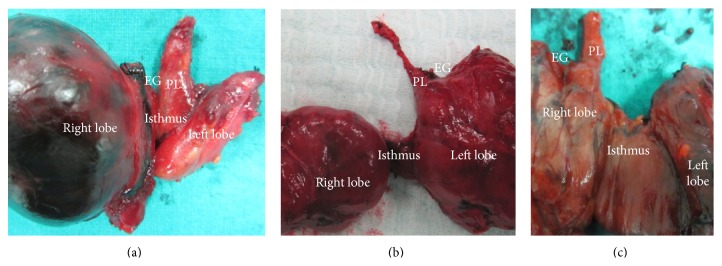
Medium pyramidal lobe (PL): (a) originating from the isthmus at the midline, (b) originating from the left lobe, and (c) originating from the right lobe.

**Figure 3:**
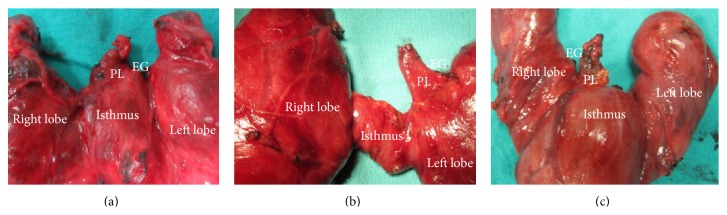
Short pyramidal lobe (PL): (a) originating from the isthmus at the midline, (b) originating from the left lobe, and (c) originating from the right lobe.

We found PLs of various lengths with origin at various sites on the thyroid gland (Figures 1, 2, and 3).

## 4. Discussion

A great majority of thyroid surgeons prefer total thyroidectomy for the treatment of malignant or benign surgical diseases of the thyroid gland. The completeness of thyroidectomy has a significant effect on appropriate surgical treatment and proper follow-up in thyroid malignancies. Moreover, incomplete excision may affect postoperative outcomes in patients with autoimmune disorders. The anatomical features of the thyroid gland create some difficulties in the total removal of the thyroidal tissue, resulting in a tissue remnant postoperatively. The PL may be considered a thyroid anomaly, a morphological variation, or a normal component of the thyroid gland [1].

If the PL is not excised during total thyroidectomy, postoperative hypertrophy of this remnant may result in recurrent disease as a midline lump even years after the primary operation. In previous studies, any functional remnant tissue was checked postoperatively by thyroid scintigraphy. The incidence of the PL remnant has been reported as 23% in a total thyroidectomy series for benign disorders, as estimated by nuclear scanning with Tc 99 m pertechnetate [2]. After a radioiodine ablation of the remnant, radioiodine neck scans have detected a remnant of thyroid tissue in 30.5% to 46% of patients who underwent thyroidectomy for differentiated thyroid cancer; nuclear scan results showed minimal residual uptake in the anterior cervical region [3–5].

Therefore, we infer that removal of the PL has considerable effect on the completeness of thyroidectomy. In the present study, we tried to establish the incidence and anatomical features of the PL in a case series of patients who underwent total thyroidectomy.

The prevalence (65.7%) of the PL in our series of 166 total thyroidectomy cases is quite comparable with that reported in previous series of surgical cases. Kim et al. [6] reported a prevalence of 59.8% in 132 patients who underwent thyroid surgery. Ryu et al. [7] have detected PL during thyroid surgery in 60% of 135 patients. Zivic et al. [8] found the PL in 61% of 100 thyroid surgeries. Series from postmortem studies have also reported the incidence of PL as being 55% to 60% [1, 9–11]. In our series, 75.2% of patients who had PLs were female. In the Cengiz et al. [12] series, 126 of 156 (81%) patients with PLs were female. In contrast, 46.9% of patients with PLs were female in Milojevic et al.'s case series [1]. Based on the findings of our study, we report that there are no gender differences in the incidence of the PL. Various results have been reported in previous case series for the prevalence of PL in men and women. Park et al. [13] reported no difference by gender; Braun et al. [9] reported that PLs were found more frequently in men than in women. On the other hand, the PL was more frequent in female (62%) than male (50%) subjects in Zivic et al.'s series [8]. The incidence of PLs was also preoperatively determined with imaging modalities to be 41.3–59.3% by computed tomography and 37.5–58.5% by ultrasound (US) [7, 13–16]. Because of the high incidence of the occurrence of the PL, thyroid surgeons should pay attention to the prelaryngeal region between the isthmus and the hyoid bone to remove all the thyroidal tissue during thyroidectomy.

From our results, we deduce that the PL predominantly originates from the isthmus, followed by origin from the left side of the midline and, uncommonly, from the right side. In approximately half of our patients, the base of the PL was located on the isthmus at or near the midline. Previous studies have also confirmed our findings that half of the PLs were located at or adjacent to the median plane [1, 8, 9]. Left of the midline was also a common location of the PL, both in the present and in the previous studies. PLs originating from the right side were uncommonly encountered during surgical and postmortem anatomical dissections [1, 11, 13, 14, 17]. Variations in the locations of the base of the PL necessitate careful dissection of the pretracheal area from the upper border of the isthmus and upper-inner borders of both the lateral lobes.

The mean length of the PL in our patients is similar to that reported in previous series. The length was reported to be between 20.13 and 22.8 in four previous articles [1, 8, 14, 15]. In three reports, the mean length of the PL is reported to be relatively longer as 25, 26.5, and 29 mm [6, 9, 13]. We found that the PL was longer in female patients than in male patients, who had a higher incidence of shorter PLs. Braun et al. [9] have reported the mean length of the PL to be 14 and 29 mm in men and women, respectively. Milojevic et al. [1] have also confirmed the prevalence of longer PLs in females than in males. In approximately three-fourths of our patients, PLs were shorter than 30 mm. Therefore, the tip of the PL was below the level of the upper border of the thyroid cartilage in the majority of our patients. Variation in the location and length of the PL generally requires dissection of the prelaryngeal region up to upper border of the thyroid cartilage in most patients and, sometimes, to the hyoid bone. We found PLs of various lengths with origin at the isthmus, the left lobe, or the right lobe of the thyroid gland as the main anatomical findings of this study.

The incidence of PL is found to be considerably higher during surgical dissection in patients with thyroid disorders.

The base of the PL is generally located on the isthmus near the midline, followed by the left side and, uncommonly, the right side. The length of the PL is variable and longer in females than in males. The high incidence and variations in the location and length of the PL may contribute to an increased risk of incomplete thyroidectomy. Therefore, during thyroid surgery, the entire prelaryngeal region between the isthmus and the hyoid bone should be dissected to excise the PL completely, if present, without leaving any remnant thyroidal tissue. Such dissection should begin at the isthmus and be advanced upward until the hyoid bone with an aim to completely remove the PL.

## Figures and Tables

**Table 1:** The incidence of pyramidal lobe in female and male thyroidectomy cases.

	Total	Woman	Man
Total thyroidectomy	166	125 (75.3)^*∗*^	41 (24.7)
Pyramidal lobe present	109 (65.7)	82 (65.6)	27 (65.9)
Pyramidal lobe absent	57 (34.3)	43 (34.4)	14 (34.1)

^**∗**^Numbers in parentheses are percentages.

**Table 2:** Location of base of the pyramidal lobe on the thyroid gland.

Location	Total	Woman		Man	
Junction of the isthmus and the right lobe or the right lobe	20 (18.3)^*∗*^	18 (90)		2 (10)	
			(22)		(7.4)

The isthmus	57 (52.3)	40 (70.2)		17 (29.8)	
			(48.8)		(63)

Junction of the isthmus and the left lobe or the left lobe	32 (29.4)	24 (75)		8 (25)	
			(29.2)		(29.6)

Total	109 (100)	82	(100)	27	(100)

^**∗**^Numbers in parentheses are percentages.

**Table 3:** The length of the pyramidal lobe.

	Length (mm)	Total	Woman		Man	
Short	1–15	42 (38.5)^*∗*^	28 (66.7)		14 (33.3)	
				(34.1)		(51.9)

Middle	16–30	42 (38.5)	34 (81)		8 (19)	
				(41.5)		(29.6)

Long	30<	25 (23)	20 (80)		5 (20)	
				(24.4)		(18.5)

Total		109	82	(100)	27	(100)

^**∗**^Numbers in parentheses are percentages.
